# KRLMM: an adaptive genotype calling method for common and low frequency variants

**DOI:** 10.1186/1471-2105-15-158

**Published:** 2014-05-23

**Authors:** Ruijie Liu, Zhiyin Dai, Meredith Yeager, Rafael A Irizarry, Matthew E Ritchie

**Affiliations:** 1 Molecular Medicine Division, The Walter and Eliza Hall Institute of Medical Research, 1G Royal Parade, Parkville, Victoria 3052, Australia; 2 Cancer Genomics Research Laboratory, SAIC-Frederick, Inc., NCI-Frederick, Frederick, Maryland 20877, USA; 3 Department of Biostatistics and Computational Biology, Dana-Farber Cancer Institute, CLSB 11007, 450 Brookline Ave, Boston, Massachusetts 02215, USA; 4 Department of Mathematics and Statistics, The University of Melbourne, Parkville, Victoria 3010, Australia; 5 Department of Medical Biology, The University of Melbourne, Parkville, Victoria 3010, Australia

**Keywords:** Genotyping, Clustering, Microarray data analysis

## Abstract

**Background:**

SNP genotyping microarrays have revolutionized the study of complex disease. The current range of commercially available genotyping products contain extensive catalogues of low frequency and rare variants. Existing SNP calling algorithms have difficulty dealing with these low frequency variants, as the underlying models rely on each genotype having a reasonable number of observations to ensure accurate clustering.

**Results:**

Here we develop KRLMM, a new method for converting raw intensities into genotype calls that aims to overcome this issue. Our method is unique in that it applies careful between sample normalization and allows a variable number of clusters *k* (1, 2 or 3) for each SNP, where *k* is predicted using the available data. We compare our method to four genotyping algorithms (GenCall, GenoSNP, Illuminus and OptiCall) on several Illumina data sets that include samples from the HapMap project where the true genotypes are known in advance. All methods were found to have high overall accuracy (> 98%), with KRLMM consistently amongst the best. At low minor allele frequency, the KRLMM, OptiCall and GenoSNP algorithms were observed to be consistently more accurate than GenCall and Illuminus on our test data.

**Conclusions:**

Methods that tailor their approach to calling low frequency variants by either varying the number of clusters (KRLMM) or using information from other SNPs (OptiCall and GenoSNP) offer improved accuracy over methods that do not (GenCall and Illuminus). The KRLMM algorithm is implemented in the open-source crlmm package distributed via the Bioconductor project (http://www.bioconductor.org).

## Background

Microarray technology has revolutionized the study of the genetics of complex disease, with large-scale case-control studies completed in the past 7 years [1,2] uncovering many thousands of new susceptibility loci for a wide range of autoimmune, mental and cardiovascular disorders and cancers [3]. These discoveries were made possible by the pioneering work of the HapMap project [4] in cataloguing genetic variation in multiple human populations. This collection has recently been expanded upon by the 1000 genomes project [5] which has enhanced the catalogue of *low frequency* and *rare variation* (defined as polymorphism with a minor allele frequency (MAF) of 0.5%–5% and < 0.5% respectively). Single-nucleotide polymorphism (SNP) microarrays with increased coverage of low frequency and rare variants are available from both major manufacturer’s Affymetrix and Illumina. Previous comparisons of genotyping methods have shown that many popular algorithms have reduced call accuracy for these SNPs [6-8]. Optimal analysis of this new content will therefore depend on calling algorithms that are capable of making sensible calls even when there are few observations, as is the case for genotypes involving the minor allele.

Illumina SNP microarrays are currently the most widely used array-based platform in both large- and small-scale genetic studies. Illumina’s largest BeadChips contain between 2.5 million and 4.3 million SNPs (Table 1) and process multiple samples in parallel (currently 4, 8, 12 or 24 per BeadChip). The Infinium II chemistry used on this platform differentially labels allele A and allele B with red and green dye respectively [9,10]. A number of algorithms are available for processing the raw signal of paired allele intensities into discrete genotype calls (AA, AB, BB) for each SNP in each sample. Current methods include: GenCall [11], Illumina’s proprietary method implemented in the GenomeStudio software; GenoSNP [12]; Illuminus [13]; CRLMM [14-16]; Birdseed [17] and BeagleCall [18]. Three new methods have been proposed recently to meet the challenge of calling low frequency/rare variants on the Illumina platform (M ^3^[7], zCall [19] and OptiCall [8]).

**Table 1:** Summary of the Illumina data sets analyzed

**Platform**	**# SNPs per**	**Data set for**	**MAF**	**# HapMap**
	**sample**	**SNP selection**		**samples**
Omni1-Quad	∼ 1.1 million	HapMap	> 5%	267 (88:44:45:90:0)
Omni2.5-Quad	∼ 2.5 million	1000 genomes	> 2.5%	171 (0:43:45:0:83)
Omni5-Quad	∼ 4.3 million	1000 genomes	> 1%	341 (172:44:40:85:0)

The number of samples from each of 5 HapMap populations (CEU:CHB:JPT:YRI:TSI) is shown in parentheses for each data set.

In this paper, we introduce KRLMM, a new genotype calling method for Illumina BeadArray data that takes a novel approach to that of other methods by allowing a variable number of clusters (*k*=1, 2 or 3) to be fitted to the between sample normalized intensity data. We analyze datasets from a number of platforms and highlight the benefit of careful signal adjustment between samples to optimize calling accuracy. We compare this approach to four existing algorithms (GenCall, GenoSNP, Illuminus, and OptiCall) by analyzing data sets with increasing coverage of low frequency/rare variants, and compare the performance of these methods in terms of accuracy at varying minor allele frequency. KRLMM is shown to perform favourably in most comparisons, particularly at low MAF.

## Methods

### Data sets

Data from HapMap samples run on 3 high density SNP platforms in-house at Illumina were analyzed (Table 1) using 5 different genotyping methods (Table 2). For each chip type, independent genotype calls (AA, AB, BB) were downloaded from the HapMap ftp server (ftp://ftp.ncbi.nlm.nih.gov/hapmap/genotypes/2010-08_phaseII+III/forward/). The number of SNPs with at least 1 non-missing call varied by chip type (851,225 for Omni1-Quad, 709,236 for Omni2.5-Quad and 1,061,706 for Omni5-Quad). These data provide us with an independent truth that can be used to compare the call accuracy of the different genotyping methods.

**Table 2:** Summary of the genotype calling algorithms compared

**Method**	**Normalization**	**Clustering model**
GenCall [11]	W	B
Illuminus [13]	W	B
GenoSNP [12]	W	W
OptiCall (v 0.6.2) [8]	W	W/B
KRLMM	B	B

Key: W - within sample method; B - between sample method.

### Signal characteristics

**Figure 1:**
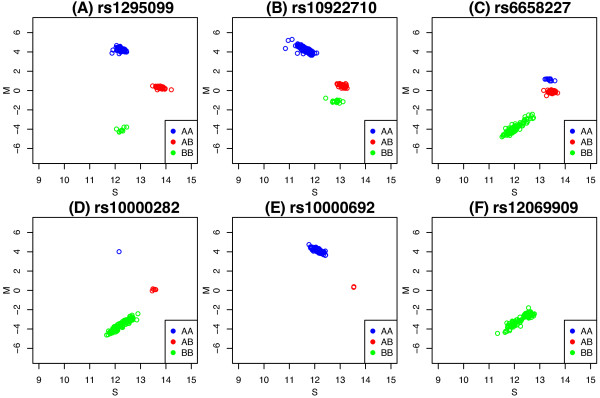
**Signal characteristics.** Plots showing typical signal for common SNPs (top row) and SNPs with lower MAF (bottom row). Panels **B** and **C** show examples of SNPs with asymmetric signal which is shifted up or down towards the heterozygous cluster. The x-axis displays average signal ($S=\frac{1}{2}[\underset{2}{\text{log}}X^{*}+\underset{2}{\text{log}}Y^{*}]$) and the y-axis the log-ratio (*M *= log_2_*X*^∗^- log_2_*Y*^∗^). The allelic signals (*X*^∗^, *Y*^∗^) have been quantile normalized as described in the Methods. The presence of 3 **(A-D)**, 2 **(E)** or 1 **(F)** distinct cluster motivates our approach to genotype calling that uses the data available for each SNP to predict the number of clusters prior to clustering. These examples are from the Omni2.5-Quad platform.

In the description below, we use Illumina’s nomenclature of *X* and *Y* to refer to the intensities of the respective alleles (in general *X *= allele A/red channel and *Y *= allele B/green channel). For well-behaved SNPs with normal copy number, log-ratios (*M *= log2*X*^∗^- log_2_*Y*^∗^) or other contrasts between the normalized intensities (*X*^∗^, *Y*^∗^) tend to be well separated into distinct clusters corresponding to the major genotypes present (Figure 1). This separation is known to vary between SNPs and can depend on the overall signal intensity. Various within and between sample modelling approaches have been proposed to adjust for these effects and convert the intensities into genotype calls, with appropriate call confidence measures (refer to Ritchie *et al.* (2011) [6] for a review).

### KRLMM algorithm

#### ***Preprocessing***

**Figure 2:**
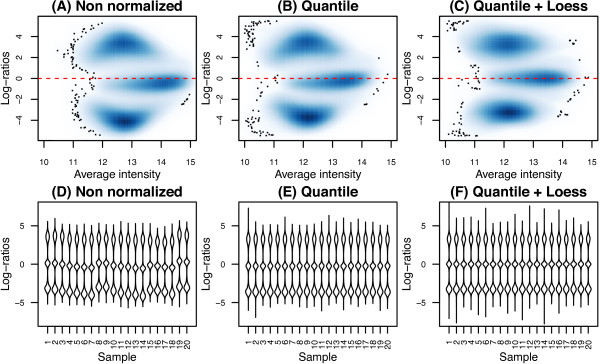
**The effect of different preprocessing methods.** Smoothed scatter plots showing data from a typical sample before **(A)** and after **(B and C)** different preprocessing methods (**B**: within-channel quantile normalization and **C**: quantile normalization (as in **B**) combined with loess correction to remove the intensity dependent curvature evident in panels **A** and **B**). In these plots, darker regions indicate a higher density of points. Panels **D** to **F** show beanplots [20] of the log-ratios from 20 samples for the different methods. Between sample consistency improves after normalization **(E and F)**. Data shown is from the Omni1-Quad platform.

Two different preprocessing methods were considered for use in KRLMM. The first involved between sample quantile normalization of each channel (*X* and *Y*) separately to adjust for systematic differences between the signal from different arrays. Although this resulted in a more consistent distribution of log-ratios between samples (Figure 2E) relative to no normalization (Figure 2D), there remained an intensity-dependent trend in the log-ratios (Figure 2B). To overcome this, an additional loess correction was applied to each major cluster (Figure 2C), with the consensus estimate of cluster center added back to the upper (AA) and lower (BB) clusters (taken as the medians of the AA and BB centers obtained after k-means clustering with *k *= 3 for each SNP).

#### ***Regression to choose k***

The KRLMM algorithm is a one-dimensional clustering method that uses k-means clustering (as implemented in the kmeans R function) for a variable number of clusters, where the choice of *k* is determined by the SNP-specific signal. As shown in Figure 1, an ideal clustering might be relatively tight (i.e. low residual sum of squares) rather than diffuse and have cluster centers with low bias when compared to the consensus positions of the AA, AB and BB clusters obtained by looking across all SNPs (as measured by the Mahalanobis distance defined below). It should also assign an appropriate number of calls to each genotype in order to obey Hardy-Weinberg equilibrium. A clustering that performs well according to these criteria is likely to be more accurate than one that scores poorly in one or more of these areas. To exploit these underlying signal characteristics and genetic principles, we applied logistic regression using these variables to predict *k* independently for each SNP.

To obtain an initial estimate of the cluster centers, k-means clustering with *k *= 3 is first applied to normalized log-ratios from each SNP separately. A vector containing the median values for the AA, AB and BB clusters (***μ***_*k*_) is used as a consensus value, and the variance-covariance matrix (${\hat{V}}_{k}$) estimated. The SNP-specific (*i*) predictors calculated across all *n*_*i *_samples (*j* is the sample index) used in the regression model include the residual sum of squares ($R_{\text{ik}}=\underset{k}{\sum }\underset{j}{\sum }{\left(M_{\text{ij}}-{\hat{\mu }}_{\text{ik}}\right)}^{2}$ for *k* clusters, where *M*_*ij *_is the normalized log-ratio), Mahalanobis distance ($D_{\text{ik}}=(x_{\text{ik}}-{\hat{\mu }}_{k})\hat{V_{k}}{\left(x_{\text{ik}}-{\hat{\mu }}_{k}\right)}^{T}$, where **x**_*ik *_is a vector of cluster centers from a given k-means clustering) and agreement with Hardy-Weinberg equilibrium ($H_{\text{ik}}=\overset{k}{\underset{l=1}{\sum }}\frac{{\left(N_{\text{il}}-n_{i}r_{\text{il}}\right)}^{2}}{\left(n_{i}r_{\text{il}}\right)}$, $r_{i1}=p_{i}^{2}$, *r*_*i*2 _= 2 *p*_*i *_(1-*p*_*i*_), *r*_*i*3 _= (1-*p*_*i*_)^2^, *N*_*i*1 _= number of AA calls made, *N*_*i*2 _= number of AB calls made and $p_{i}=\frac{2N_{i1}\;+\;N_{i2})}{2n_{i}}$ is the empirical major allele frequency based on a given number of clusters, *k*). Each variable is calculated for each SNP using the cluster assignments obtained via k-means clustering with *k *= 1,2,3 (agreement with Hardy-Weinberg equilibrium was not calculated for *k *= 1 as this quantity is not informative) leaving 8 variables in the regression. Coefficients for each parameter were estimated from fitting this model to a training set of 10,000 randomly chosen SNPs from the HapMap data sets. The independent genotype calls provide us with the true *k* for these SNPs. Both ordered logistic regression (assumes that the groups *k *= 1,2,3 are ordered, consistent with increasing dose (0, 1 or 2 copies) of the alternate allele), or regular logistic regression (no ordering assumed) as available in the polr (MASS R package) and vglm functions (VGAM R package) respectively were used. Once regression coefficients are available, and given a complete set of covariates (*R*_*ik*_, *D*_*ik*_, *H*_*ik *_for all *k*), the best *k* is determined for each SNP by obtaining fitted values from the model and choosing the *k* with maximum probability. Next k-means clustering is applied using the predicted value of *k* to obtain genotype calls. This approach is applied to all SNPs from autosomes and pseudo-autosomal (XY) regions of the genome.

#### ***Call confidence measures***

The silhouette width (${\text{SW}}_{\text{ij}}=\frac{{\hat{b}}_{\text{ij}}-{ŵ}_{\text{ij}}}{\text{max}({ŵ}_{\text{ij}},{\hat{b}}_{\text{ij}})}$, where ${\hat{b}}_{\text{ij}}$ is the smallest average *between* cluster distance for the *ij*th observation and all other observations in a different cluster and ${ŵ}_{\text{ij}}$ is the average *within* cluster distance for the *ij*th observation and all other observations from the same cluster) as calculated in PAM clustering [21] is used as a call confidence measure. This value will be near 1 for calls made with high confidence (${ŵ}_{\text{ij}}$ will be small relative to ${\hat{b}}_{\text{ij}}$) and -1 for low certainty calls (where ${ŵ}_{\text{ij}}$ is large relative to ${\hat{b}}_{\text{ij}}$ and dominates the calculation).

#### ***Choosing an optimal Preprocessing/Regression combination***

**Figure 3:**
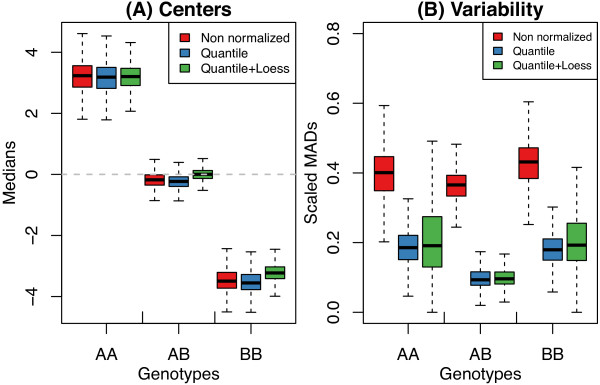
**Impact of preprocessing on signal and noise.** Plot showing the cluster centers **(A)** and variability **(B)** after various pre-processing methods have been applied. Values were obtained using the independent genotype calls available from the HapMap database for the majority of SNPs. Quantile with loess, which we saw in Figure 2C removes intensity-dependent curvature from the log-ratios, does so at the cost of increasing the variability for some homozygous calls. Results plotted are from the Omni1-Quad platform.

**Figure 4:**
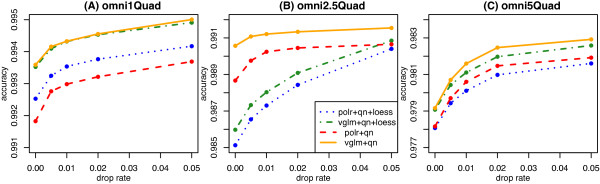
**Accuracy versus drop rate for various combinations of preprocessing and regression analysis for predicting *k *.** Results are shown for the Omni1-Quad **(A)**, Omni2.5-Quad **(B)** and Omni5-Quad **(C)** platforms. The combination with the highest accuracy is quantile normalization coupled with regular logistic regression (solid gold line). Other combinations, such as quantile normalization with loess adjustment and ordered logistic regression (dotted blue line), or quantile with ordered logistic regression (dashed red line) and quantile with loess adjustment and regular logistic regression (dashed green line) are either generally less accurate, or perform inconsistently between platforms. Accuracy (y-axis) refers to concordance with independent HapMap calls and drop rate (x-axis) is the proportion of calls removed due to low call confidence as measured by silhouette width.

Using the independent HapMap calls, the median and median absolute deviation (MAD) of each cluster (AA, AB, BB) was determined to obtain a SNP-level view of the effect of normalization (Figure 3). Although the centers were more consistent after quantile adjustment with an extra loess correction compared to quantile normalization alone (Figure 3A), the effect on the variability was a mixed one for homozygous clusters, with some clusters becoming more variable, relative to no normalization and some less variable (Figure 3B). Quantile normalization generally lowered the between sample variability more consistently than no normalization and quantile normalization combined with loess correction.To assess the performance of the different regression/preprocessing combinations, the concordance between the genotype calls made by each method were compared with the independent calls obtained from the HapMap database. Figure 4 shows the results from the two logistic regression methods (ordered logistic regression in polr and regular logistic regression in vglm) coupled with either quantile normalization alone or quantile normalization followed by loess adjustment. The drop rate refers to the proportion of SNPs that have been removed from the accuracy calculation on account of low call confidence measures. For each data set, regular logistic regression applied to log-ratios after quantile normalization gives higher accuracy. This optimal preprocessing combination was chosen as the default implementation of KRLMM in the remainder of this paper.

#### ***Calling SNPs from sex chromosomes***

SNPs outside of pseudo-autosomal regions on the X and Y chromosomes represent special cases that are called separately for males and females to allow for the appropriate number of clusters (Y chromosome: No call for females, *k *= 1 or 2 clusters are permitted in males since they have 1 copy of this chromosome. X chromosome: *k *= 1,2,3 clusters are allowed for females, since they have normal copy number, and *k *= 1 or 2 is allowed for males since they are hemizygous). When gender (male/female) of the samples is not specified, the average intensities (*S*, x-axis in Figure 1) from the Y chromosome SNPs are used to impute this information. Applying k-means clustering with *k *= 2 clusters separately to all chromosome Y markers should assign females to the low signal group (i.e. background hybridization only from these probes since females don’t have a Y chromosome) and the males to a second high intensity group that corresponds to signal from one copy of the Y chromosome. Samples are then assigned as female or male by looking at which cluster (low intensity = female, high intensity = male) is closer on average by comparing the median cluster position determined by k-means and the median *S*-value for these SNPs within each sample. This simple approach was found to be 100% accurate on each of the 3 platforms upon comparison of the imputed gender with the information provided by the HapMap database.

#### ***Implementation***

**Figure 5:**
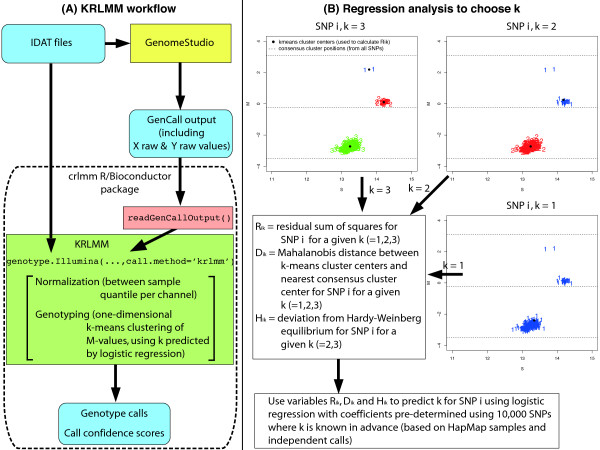
**Summary of the KRLMM algorithm.** Analysis begins with either idat files or output from GenomeStudio. Data are then processed by the genotype.Illumina function to generate genotype calls and call confidence scores **(A)**. A key part of the KRLMM algorithm is determining the number of clusters (*k *= 1,2, or 3) for each SNP **(B)**. Variables that measure the tightness of a particular clustering (*R*_*ik *_for *k *= 1,2,3), the amount of bias present in the estimated cluster positions (*D*_*ik *_for *k *= 1,2,3) and agreement with Hardy-Weinberg proportions (*H*_*ik *_for *k *= 2,3) based on k-means cluster assignments for different values of *k* are calculated for each SNP. In each plot, points have been numbered and colored according to the k-means clustering results for a given *k*. Regression coefficients for each of these variables are pre-determined (and saved in platform specific annotation packages) by fitting a logistic regression model to a training data set made up of 10,000 randomly chosen SNPs from a HapMap data set where the genotypes (and true *k*) are known in advance. This model is applied to the SNP-specific predictors to determine the best *k* to use in a k-means clustering to obtain genotype calls.

An overview of the KRLMM algorithm and the regression analysis used to determine *k* for each SNP is presented in Figure 5. The genotype.Illumina function available in the crlmm package [15,16] from R [22]/Bioconductor [23] implements this approach when call.method=~krlmm~, allowing users to read in IDAT files using capability available through the illuminaio package [24] and make genotype calls in one simple command. A second option for importing data is offered through the readGenCallOutput function, which handles either tab or csv delimited GenomeStudio reports. Regression coefficients and reference distributions for the quantile normalized *X* and *Y* channels obtained from analyzing Illumina’s in-house HapMap samples are stored in chip-specific data packages also available from Bioconductor (http://www.bioconductor.org). The KRLMM method does not currently support data from Affymetrix genotyping arrays.

### Other algorithms

Table 2 summarizes the major features of the 5 algorithms compared in terms of normalization method and underlying model. Normalization occurs either within (GenCall, Illuminus, GenoSNP, OptiCall) or between sample (KRLMM). Likewise, the model-based clustering can occur within sample (GenoSNP), between sample (GenCall, Illuminus, KRLMM) or in a hybrid manner (OptiCall). For a review of the first 3 methods, see Ritchie *et al.* (2011) [6].

The OptiCall algorithm is a hybrid between GenoSNP and Illuminus. It first makes use of data from 50,000 randomly chosen intensity values to do an initial clustering using a mixture model with 4 states (AA, AB, BB and  NoCall’). This step is akin to GenoSNP’s between SNP approach for genotyping. OptiCall then clusters SNP-by-SNP using a model similar to the one used in Illuminus, with added hierarchical structure including priors derived from the initial clustering. In clusters with few observations, the prior ensures the clusters are sensibly placed, thus overcoming one of the shortcomings in Illuminus. We chose OptiCall as a representative method from the newer calling methods tailored for low frequency/rare variants. M ^3^[7] was also considered, however its implementation in MATLAB (which requires a licence) precluded us from using this software. Another option is the zCall algorithm [19], which unlike the other methods compared, begins with the output of GenCall rather than the raw intensity data. Such a post-calling correction method could presumably be adapted to improve the calls from any method once calls and confidence values are available from the raw data.

## Results and discussion

**Figure 6:**
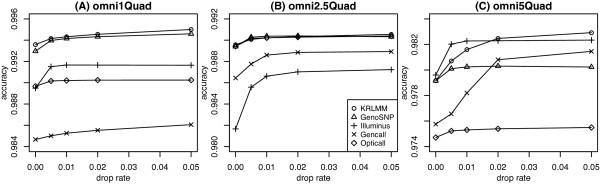
**Accuracy versus drop rate for 5 genotyping methods.** Results are shown for the Omni1-Quad **(A)**, Omni2.5-Quad **(B)** and Omni5-Quad **(C)** platforms for each genotyping method (autosomal SNPs only). KRLMM tends to have the best or equal best performance amongst the methods compared.

**Figure 7:**
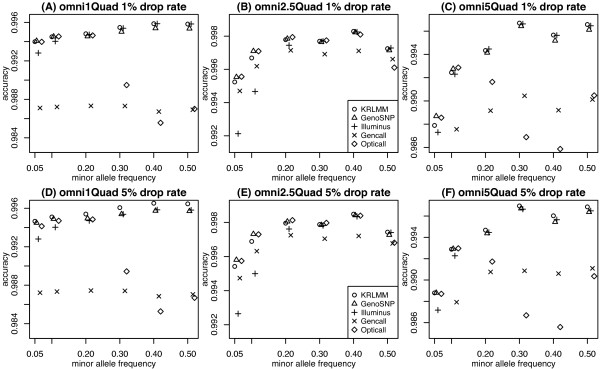
**Accuracy versus MAF at different drop rates for common variants.** Results are plotted for the Omni1-Quad **(A and D)**, Omni2.5-Quad **(B and E)** and Omni5-Quad **(C and F)** HapMap samples for each method at drop rates of 1% (top row) and 5% (bottom row) for autosomal SNPs only down to a MAF of 5%. The accuracy of GenCall at MAF of 5% (0.05) is slightly below the lower limit shown in the plot at 0.983 in panel **C** and 0.984 in panel **F**.

For performance comparison, concordance between the genotype calls made by each method and the independent calls obtained from the HapMap database were used to calculate accuracy. Figure 6 shows the accuracy of each method for the 3 data sets listed in Table 1 for autosomal SNPs at varying drop rates. Overall differences between the five methods are modest, with most delivering accuracy above 98%. KRLMM has the best or equal best performance amongst the methods compared, while GenCall tends to have lower to intermediate accuracy.The performance of OptiCall varies the most between data sets: in the Omni5-Quad data set (Figure 6C) it is marginally worse than all other methods; in the Omni1-Quad data set (Figure 6A) it is more accurate than GenCall, but less accurate then the other methods (KRLMM, GenoSNP and Illuminus) while in the Omni2.5-Quad data set (Figure 6B) its accuracy is comparable to the other methods. Given OptiCall’s hybrid approach between GenoSNP and Illuminus, this inconsistent behaviour was surprising, with performance on par with these two methods expected. Although overall differences in accuracy between the methods are modest, given the large size of the respective data sets, differences as small as 0.5% will translate to thousands of additional correct genotype calls per sample. For example, the difference between KRLMM and GenCall in Figure 6A at a drop rate of 2% equates to an average of 7,103 additional correct calls per sample. In Figure 6C at the same drop rate (2%), the difference in accuracy between KRLMM and OptiCall equates to an average of 6,217 additional correct calls per sample.We next stratify accuracy by MAF and concentrate on common SNPs (those with MAF > 5%, Figure 7). Ignoring the overall differences in accuracy that are consistent with the results seen in Figure 6, we see that the performance of different methods varies by MAF. For the majority of methods, accuracy increases gradually with MAF, consistent with the idea that the clustering problem becomes easier as the number of observations in the clusters involving the minor allele increases. OptiCall is an exception to this trend, giving less accurate calls for common SNPs than Illuminus; this is especially pronounced for the Omni1-Quad (Figure 7A and 7D) and Omni5-Quad chips (Figure 7C and 7F). This highlights the reason for the poorer overall performance of OptiCall relative to Illuminus and GenoSNP for these chips (Figure 6A and 6C). The accuracy is only marginally worse (∼ 1%) for OptiCall compared to other methods, however given the ease at which SNPs with higher MAF can be called by other algorithms, it may suggest the presence of a minor bug in the version of the software we used (version 0.6.2). Differences between the methods are consistent as the number of low confidence calls removed increases from 1% (Figure 7, A-C) to 5% (Figure 7, D-F).

**Figure 8:**
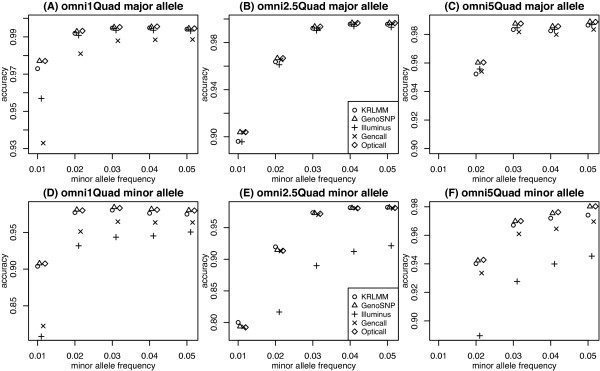
**Accuracy stratified by major/minor allele for low frequency variants.** Calls involving the major allele only (top row) or minor allele (bottom row) from low frequency SNPs (MAF < 0.05 (5%)) in Omni1-Quad **(A and D)**, Omni2.5-Quad **(B and E)** and Omni5-Quad **(C and F)** HapMap samples for each method at a drop rate of 1%. The accuracy of some methods falls below the lower limit plotted in each panel. These values are as follows: panel **(C)** MAF 0.01: KRLMM 0.796, GenoSNP 0.808, Illuminus 0.789, GenCall 0.799, OptiCall 0.809; panel **(E)** MAF 0.01: Illuminus 0.607; panel **(F)** MAF 0.01 KRLMM 0.707, GenoSNP 0.706, Illuminus 0.624, GenCall 0.686, OptiCall 0.708.

**Figure 9:**
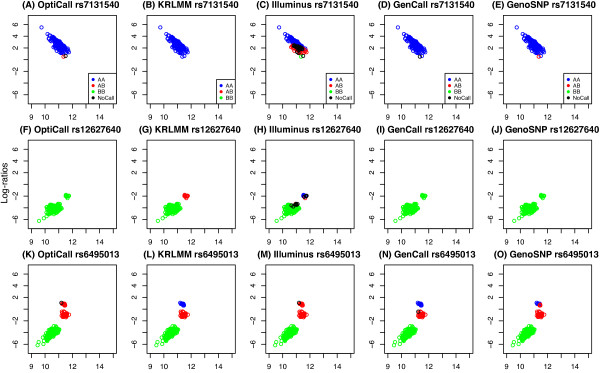
**Examples of SNPs where KRLMM has improved accuracy over other methods.** Plots presenting log-ratios (*M*-values) versus average expression (*S*-values) for 3 SNPs where KRLMM has perfect agreement with calls from the HapMap database, and the other methods have some level of disagreement are shown **(A-O)**. In each plot, points are color-coded according to the genotype call made by the particular method.

We next zoom in and focus on the ability of different genotyping methods to call low frequency content (SNPs with MAF < 5%). Accuracy was stratified to look at calls involving either the major allele only or the minor allele. This is a key comparison, as methods with a systematically higher error rate for calls involving the minor allele will pay a negligible price in terms of overall accuracy since there are so few of them. Figure 8 plots results by major (panels A-C) and minor (panels D-F) allele for each method at a drop rate of 1%. For calls of the major allele, all methods show comparable accuracy (Figure 8, A-C). GenCall is slightly less accurate for the Omni1-Quad platform (Figure 8A), but this can be attributed to use of an older version of GenCall (GSGT v 1.1.9 for Omni1-Quad versus GSGT v1.6.3 for Omni2.5-Quad and v1.8.4 for Omni5-Quad). For minor allele calls (Figure 8, D-F), Illuminus is generally slightly less accurate, followed by GenCall in the Omni1-Quad and Omni5-Quad data sets (Figure 8D and 8F). KRLMM performs comparably to OptiCall and GenoSNP across the board for both the major and minor allele calls. In particular for the low frequency content (MAF 0.01) on the Omni5-Quad platform (Figure 8F), KRLMM, GenoSNP and OptiCall all have accuracy above 0.70, whereas GenCall and Illuminus are below this level (0.686 and 0.624 respectively). As previously noted, the performance of Illuminus improves as more samples are available [6], with higher accuracy achieved on both the Omni1-Quad (267 samples) and Omni5-Quad (341 samples) compared to the Omni2.5-Quad data set (171 samples). This highlights the difficulty faced by methods that model 3 clusters for every SNP using only the data at hand. Clusters with no or few observations, as occurs at low MAF, are more likely to be erroneously called. As sample size increases, there is more data to support these minor clusters, which improves the accuracy of Illuminus to a level comparable to that of other methods. The accuracy of OptiCall is on par with GenoSNP as expected, since OptiCall makes use of priors obtained from other SNPs, thereby overcoming the major limitation of Illuminus.To explore situations where the adaptive approach of KRLMM is beneficial, we examined SNPs where KRLMM is in perfect agreement with the calls from the HapMap database and the other methods make varying numbers of mistakes. Figure 9 shows 3 examples that exemplify the main categories observed: SNPs with fewer than 3 clusters (top and middle rows), SNPs with less well separated clusters (bottom row, as compared to Figure 1A and 1D), or SNPs with signal that is skewed towards one of the homozygous clusters (middle and bottom rows). The first situation will benefit from KRLMM’s adaptive approach to clustering, while the presence of shifted signal shows the utility of SNP-specific clustering over the more global approaches of GenoSNP and OptiCall that cannot accommodate skewed cluster positions.

## Conclusion

Overall, the KRLMM algorithm marginally outperforms the vendor’s own alternative (GenCall) in terms of accuracy, even at low minor allele frequencies. For low frequency variants, our results show the merit of approaches that use the signal from other SNPs either directly in their models (OptiCall, GenoSNP) or indirectly to help choose the appropriate number of clusters (KRLMM) to give more accurate calls. Such approaches offer modest improvements over GenCall and Illuminus that do not have a tailored approach for dealing with SNPs with low MAF.

The KRLMM method uses k-means clustering of the log-ratios obtained after between sample quantile normalization of the raw intensities and logistic regression to determine the number of clusters to fit to each SNP. It is the only algorithm to date that allows flexibility in the number of clusters. Although the data we have used to benchmark our approach (generated in-house at Illumina) is known to be of very high quality, we are still able to detect small performance differences between our method and the vendor provided alternative. These performance differences are expected to be more pronounced in disease association studies, where samples are collected over an extended period and will be subject to additional sources of variation. The benefit of KRLMM’s between sample normalization is anticipated to deliver additional performance gains in such settings.

The KRLMM method requires a small subset of training data (i.e. 10,000 SNPs with known *k*). Although this information was obtained from HapMap data in our case, the regression coefficients could just as easily be estimated using calls from another method if such data were more readily available, as may be the case for platforms from other species. Support for newer Illumina platforms can be easily added and future extensions of the method to handle data from the two-color Axiom platform from Affymetrix are also conceivable. Another adaptation would be to allow *k *> 3 so that KRLMM could be used on cancer samples where gains and losses in the genome introduce copy number variation.

Future comparisons of the performance of different genotyping algorithms on data from a genome-wide association study (GWAS) or suitable non HapMap control data set, such as the large collection of reference samples in Wang *et al.* (2011) [25] would also be instructive. Previous work has demonstrated that the choice of method can influence GWAS results obtained from the Affymetrix platform [26]. This important extension will determine whether the same holds true for studies that use Illumina SNP arrays. Publications on this platform to date predominantly rely on calls from the GenCall or Illuminus algorithms. This will be of particular interest at the rare or low frequency end of the minor allele spectrum, especially when it comes to any significant associations detected or missed by particular genotyping methods. Access to a suitable reference GWAS data set where raw data for both cases and controls is available will be the key to such a comparison.

## Abbreviations

GWAS: Genome-wide association study; KRLMM: The new genotype calling algorithm described in this article; MAF: Minor allele frequency; SNP: Single-nucleotide polymorphism.

## Competing interests

The authors declare that they have no competing interests.

## Authors’ contributions

RL, MR and RI devised the algorithm and ZD, RL and MR implemented the method in public software. MY provided advice on algorithm development and data sets. RL and MR performed data analysis and MR wrote the manuscript with input from RL and RI. All authors read and approved the final manuscript.
